# New York Odontological Society

**Published:** 1894-02

**Authors:** 

**Affiliations:** New York Odontological Society


### Reports of Society Meetings.

### NEW YORK ODONTOLOGICAL SOCIETY.

A REGULAR meeting of the New York Odontological Society was held on Tuesday evening, November 21, 1893, at the New York Academy of Medicine, No. 17 West Forty-third Street, Dr. Woodward presiding.

The minutes of the previous meeting were read and approved.

Under the head of "Incidents of Office Practice and Casual Communications," Dr. Van Woert exhibited a mouth-lamp which was much less expensive than the ordinary electric mouth-lamp used and just as effective.

*The President.*—At our last meeting Dr. Van Woert, of Brooklyn, presented a paper on "Pyorrhœa Alveolaris," and the Executive Committee thought it well to continue the subject for this evening. Professor C. N. Peirce, of Philadelphia, has come prepared with a paper, which he will now read to us.

(For Dr. Peirce's paper, see January number, page 1.)

Dr. Peirce followed the reading of the paper by the subjoined remarks:

Frequently patients come designating a certain tooth, which, they state, was perfectly sound and firm until such a person wedged or hammered it. "Now see its condition!" The theory taken by pathologists is that the urates are not in any tissue until that tissue has had its nutrition disturbed. I had a circumstance occur about two weeks ago in my own practice which I think has given me some light. A gentleman who lost all his teeth from pyorrhœa, and who for some years suffered with what he thought was paralysis of the pharynx, finally had an operation for calcic deposit in the bladder, when two large stones were removed. His son is a patient under my care at this time. Two weeks ago I discovered a small cavity on the distal surface of an inferior molar. I packed between the molars some cotton to separate them, and renewed it once or twice, and then filled the cavity. He has been in three or more times complaining of pain in the filled tooth, which has since developed pyorrhœa. For three nights it had awakened him. That the tooth was free from pyorrhœa until very recently I have no doubt. My theory is that I must have produced a certain amount of irritation and disturbance of nutrition in the tissue at the end of the root, and by doing so made it susceptible to a ready receptacle of the uric salt and the subsequent consequences. That is the only explanation I can give which is consistent with the theory now held by pathologists.

I have brought with me some pyorrhœa specimens, also some which I have termed ptyalogenic, the former being hæmatogenic. When uric acid deposits are broken up by heat we have ammonia set free, and it exhibits itself by coloring the litmus-paper blue. There is, however, the danger of having organic matter decomposed at the same time, which will also set free ammonia and give a similar result.

DISCUSSION.

*Dr. Jarvie.*—Will you please give us the two terms again?

*Dr. Peirce.*—The terms I had prepared were ptyalogenic calcic pericementitis and hæmatogenic calcic pericementitis.

I have here some of the deposit taken from this root. I will put it in this tube and place the litmus-paper, moistened by distilled water, in the neck of the bottle. Now, by subjecting it to heat, you see that the paper is changed to a decided blue color.

*Dr. Van Woert.*—Before Professor Peirce finishes, I wish he would give us a little idea of his treatment of this disease.

*Dr. Peirce.*—I was a little premature in my paper. I should have had another month before I read it to you, but as the meeting was at this date, I gave it in an unfinished condition. I have only two patients now on constitutional treatment, and therefore I cannot give any statements regarding its success; but I have a number of patients who come to me regularly, and I shall take the most favorable opportunity to place them under the treatment suggested in the paper, to see whether I can accomplish anything.

*Dr. Van Woert.*—I mean as to the surgical or mechanical treatment.

*Dr. Peirce.*—I simply use the scalers in my best efforts to cleanse the roots, considering the deposit an irritant; then I treat with a good antiseptic, and the most satisfactory to me is aristol dissolved in chloroform, with the addition of oil of gaultheria and oil of cinnamon or cassia, equal parts of each.

*Dr. Allen.*—But that does not dissolve the deposit.

*Dr. Peirce.*—No; it is merely an antiseptic. To cleanse the root, I make an application of trichloracetic acid with great care, for it is a powerful caustic.

*Dr. Hodson.*—Do you use that in the pockets?

*Dr. Peirce.*—Yes; whenever the patient comes to the office for treatment, I wash out the pocket with peroxide of hydrogen, and then make this application followed by the antiseptic. I have found no local treatment more satisfactory. If the gums are much congested on the surface, I paint with tincture of iodine in addition.

*Dr. Jarvie.*—Is there a breaking down of the tissue?

*Dr. Peirce.*—Yes, of course; the inflammatory condition with exudation degenerating into pus facilitates this.

*Dr. Perry.*—In your paper you gave some prominence to the presence of micro-organisms as being one of the factors in producing this condition.

*Dr. Peirce.*—I said the bacteria present had been considered by Dr. Black as pathogenic, but the pathologists do not consider them so. The staphylococcus is not considered a pathogenic bacteria, although Dr. Black thinks it is. He says he can take the pus of pyorrhœa and inoculate sound or healthy tissue and produce a similar condition. He will inoculate, of course, on the gingival borders, and he may produce an irritation, but I do not think he can produce true hæmatogenic calcic pericementitis. Whatever irritation he establishes in this way is amenable to local treatment.

*Dr. Perry.*—You use your remedies in an antiseptic manner, and that is an assumption that bacteria are one of the factors.

*Dr. Peirce.*—Yes, they are a factor in degeneration. I use it to prevent their further influence in the breaking down of the soft tissue.

*Dr. Hodson.*—Does Dr. Peirce expect with these patients to merely fill the pockets anew every month, or is it necessary to go through and examine the whole socket to find out if there are any calcic deposits?

*Dr. Peirce.*—I make the examination of the teeth every month. As far as I can, I remove the deposits without lacerating the soft tissue, and then wash out with peroxide of hydrogen and apply the preparation of which I have spoken.

*Dr. Allen.*—Did you say you had found these deposits while the gingival border of the tooth was intact and perfect?

*Dr. Peirce.*—I have had cases which I have so interpreted. These cases have subsequently developed into pyorrhœa, and I have been confirmed in that by carefully watching them. A deposit will take place at the end of the root with exudation as a concomitant while the gingival borders are intact, the exudation degenerating into pus, and this following the line of least resistance finds exit at the gum margins.

*Dr. Allen.*—In one part of his paper the essayist places us in a position between "the devil and the deep sea." In other words, he said the process of separating the teeth, by producing an irritation or an abnormal condition of the peridenteum, would produce a deposit of the calcium urate; that the wedging of the teeth would leave the membrane in a condition that was susceptible to this deposit of lime salts. I would like to know exactly how far he would carry that, in advising us to alter or amend our practice in regard to separating teeth for filling.

*Dr. Peirce.*—I said pathologists had stated that the deposit of a urate was not made in a tissue unless the nutritive conditions of that tissue were somewhat disturbed. A patient of whom I have spoken was of a uric-acid diathesis, inherited from his father. This patient, being of such a diathesis, I believe that I induced this condition by wedging the tooth. I have had in my practice patients come to me and say, "Look at my front tooth. It was perfectly healthy until such a person wedged it." A lady recently brought a tooth to me in her hand, and said, "That tooth was sound until a dentist wedged it, and then there was pus around it, and it came out." I examined her mouth and found she had pyorrhœa. Dr. J. A. Woodward, of Philadelphia, asked me a short time since if we did not induce pyorrhœa by ligatures around the teeth. I did not quite appreciate his question then, but now I can see it if it is true that the deposit is not made in healthy tissue, but in tissue where the nutrition is disturbed. In a patient with a uric-acid diathesis we may induce pyorrhœa by a ligature or by causing an irritation in any other manner.

*Dr. Perry.*—What is to be said and thought, if this theory be true, of a certain number of cases that we meet, where teeth loosen and come out without any signs of calcic deposit?

*Dr. Peirce.*—It is simply the fact that the urates may be carried out by the pus and not deposited. The tooth the lady brought me last week was perfectly free of deposit, and yet pus had flowed freely from it for over a year. The flow of pus and the inflammation of the pericemental membrane which induces the absorption of the process would, I take it, be indications of the pyorrhœal condition.

*Dr. Howe.*—I can hardly express my gratification and pleasure at listening to this paper from Dr. Peirce. At the last meeting I opposed the view that micro-organisms were an important factor in the causation of this disease, and said, also, I could not accept the opinion that calcic deposit was essential to the origin of the lesion, because the disease may begin and progress without any deposit being discoverable beneath the gum margins. It gives me great satisfaction to listen to this record of Dr. Peirce's original investigations and the proofs he has to offer that this trouble is—what I have long believed—of constitutional origin. I would object, however, to his terminology for the reason stated, that solid concretions are not necessarily connected with the form of the disease which he has well called hæmatogenic. The word calcic I think is misleading, and ought not to be used in such connection. I think it probable that he believes as I do, that the deposit is a factor in aggravating the disease and in preventing restoration of the tissues to health, and that therefore, as far as possible, it ought to be removed; but that it is not an etiological factor. I said the other night that I had seen many cases of pyorrhœa in which there was no calcic deposit concerned in the destructive progress, and one or more gentlemen said, to my surprise, that they had never seen such a case. Yesterday and to-day, while treating two cases which have been in my hands for some time, I took impressions of teeth about which characteristic shrinkage of the gum has taken place: a shrivelling of the gum at and above the margin, but no recession has occurred. The gums are anæmic and thin, and the loss of part of the alveolar borders has caused the gums to sink down, so that a crease or wrinkle can be seen along the line of the loss of hard tissue under them. There is slight loosening of the teeth, perceptible to the fingers, but there are no concretions under the gums. I will pass these models around. I believe that these cases, although not so common, are just as characteristic of the disease as if there was ever so much calcic deposit. There is no doubt in my mind that all these troubles come from constitutional causes. I have been talking to my patients on that line for a good while.

In February of 1891, on my relating to Dr. T. F. Allen, a well-known physician of this city, the characteristics of this disease, as well as of erosion of the teeth, he stated it to be his belief that all such destructive processes were due to lithæmia. He said that if treatment, diet, and so forth were prescribed which were adapted to a condition of excess of uric acid in the system, these local lesions would be favorably affected and possibly cured. Shortly after that, questioning a patient who had considerable erosion, mostly affecting the roots of the teeth, which were exposed to a great degree by wasting of both alveoli and gums, some teeth being loose, she said she was gouty by inheritance from both parents, and that Dr. Allen —before referred to—had told her more than ten years before that the condition of her teeth and gums was due to her gouty tendency. Since that time I have been observing and questioning all patients who have either erosion or pyorrhœa alveolaris, or waste of alveolar and gingival tissues without distinct pyorrhœa, and have been quite convinced that all of these lesions of dental tissue are associated with the lithæmic condition. I therefore congratulate Dr. Peirce on being the first to publicly make known what I believe to be the truth in regard to the etiology of pyorrhœa. Of course there is much to be done in elucidating the theory. I have made some observations which I hope to be able to present at some future time.

*Dr. Jarvie.*—What proof have you that there is no calcic deposit on those roots?

*Dr. Howe.*—The usual proof. Failure to find it.

*Dr. Jarvie.*—There is nothing on the model to indicate that there is not.

*Dr. Howe.*—All those teeth are marked with a characteristic shrinkage of the gum.

*Dr. Jarvie.*—I should say from the model that you would find the calcic deposits between the second molar and wisdom-tooth.

*Dr. Howe.*—What would you say about the other molars?

*Dr. Jarvie.*—I do not see any great recession of the gum.

*Dr. Howe.*—That is just the point. There is no recession of the gum. There is a peculiar depression and thinning of the gum tissue, caused by the destruction of the alveolar margins, under the soft tissue. All these molars are affected in the same way. The destructive process has begun, and advanced sufficiently to cause some of them to be slightly loosened, but there is neither recession of the gum nor concretions under the gum. The real condition is only revealed by careful observation.

*Dr. Walker.*—In regard to the patient in whose mouth Dr. Peirce placed the wedge: do not we use that same pressure in correcting irregularities of the teeth?

*Dr. Peirce.*—Yes; always in moving the teeth.

*Dr. Walker.*—If this theory be true, would it not be well in extensive cases of correction to make a thorough examination of the patient before we undertake these cases?

*Dr. Peirce.*—It is very fortunate that in regulating cases our patients are invariably less than thirty years of age. This disease is not known to be developed except in very rare cases until after thirty or thirty-five years of age. I think the physicians will tell you that as a usual thing local expressions indicating the uric-acid diathesis are not established until after thirty-five or forty years, and therefore we would not be likely in our young patients for whom we correct irregularities to find any such condition.

*Dr. Walker.*—I would like to ask Dr. Jackson, who has had extensive practice in such cases, if he finds that by the movement of the teeth, either rapid or slow, that he gets this trouble?

*Dr. Jackson.*—I have not observed ill effect from moving the teeth in any case of irregularity I have corrected, although I have seen cases with extensive recession and pockets under the gums, where ligatures and rubber bands have been used by other practitioners. I may be better able to answer the question in the future, as I am now regulating the teeth for several older patients, one a lady thirty-eight years of age, for whom I am moving outward the four superior incisors which were erupted inside of the lower arch.

I have been much pleased and interested with the paper presented this evening, and think it is in the true line in which we should investigate. I am especially pleased because I have been trying to trace out the relationship of the systemic and local conditions, and have not, I find, followed the true line of investigation.

A few months since I removed three teeth from the mouth of a lady about twenty-three years of age that were so loose from pyorrhœa alveolaris that they were becoming extremely irregular and unsightly, and it was not thought advisable to correct the irregularity. A permanent fixture to retain them would have been required. On removing the teeth the roots were found to be especially short. The father of the patient is now suffering with heart and kidney trouble, and one of the sisters has pulmonary trouble and other complications. She is about twenty-two years of age. I have been interested in tracing the histories of these cases.

*Dr. Perry.*—Some time since, in reading a short paper on the subject of erosion before this Society, it may be remembered that Dr. Bulkley in his discussion of that paper, laid great stress on the great diversity of conditions that he considered due to an excess of uric acid in the blood, or what is generally called the uric-acid diathesis. Gentlemen who are more or less familiar with the drift of medical literature at the present time may have noticed that physicians are impressed by the diversity of conditions due to this excess of uric acid. This idea as applied to the teeth seems to me a very probable one. It has much to strengthen it. It is an inherited disease. I think we are all quite sure of that. We know that a gouty disease is a disease of middle life or old age, and that it is an inherited condition. One or two other points confirm my notion of this. We know that gout is curable by conforming to certain hygienic conditions, although the tendency to it cannot be eradicated. It has been said that this disease of the gums is not curable, but I remember one or two cases where the condition of the mouth has been vastly improved, and there was no explanation for it except change of condition and change of life. One patient who had this condition with three or four front teeth moved from New York and went to the interior to live. I did not see her until three or four years afterwards. When she came to me her teeth were vastly improved. They were almost well, and yet they had had no treatment during that time. She was a lady in middle life. This case would indicate that the change of scene and change of diet had benefited her, and whatever was the condition, it had been eliminated and her teeth had improved.

As far as the treatment is concerned, I think that the antiseptic treatment will be found to be the most desirable of all. It is a great point gained that Dr. Van Woert, in starting this question and Dr. Peirce in following it out so scientifically, should have put us on a new track, so that this hacking and digging at the margins of the bones will be partly checked, whatever may be the real truth of the theory.

*Dr. Starr.*—I would like to ask Professor Peirce whether he applies the term "ptyalogenic calcic pericementitis" to what we generally call "salivary calculus," where the deposits collect in great quantities and cause destruction of the teeth. If he does apply it to such cases, I would like to know the reason for giving the term "calcic pericementitis." Why not as well call it gingivitis as pericementitis?

*Dr. Peirce.*—Yes, I do so apply it. The term gingivitis would be very appropriate if the irritation were confined to the gums; but from irritation of continuity the periosteum becomes also involved, and this produces an absorption of the process as well. We have the irritation of the periosteum on both sides of the process. Of course it is then absorbed very rapidly until it is all destroyed and the tooth removed. I have many cases on the table where the tooth has been removed by the encroachments of these ptyalogenic or salivary deposits.

*Dr. Van Woert.*—I am more than gratified that Dr. Peirce bears me out in my statement, that necrosis is never present in this disease. My investigations have been to find the true local, and not the constitutional conditions, and am a little disappointed that he has said nothing of his treatment, and particularly did I wish to know more of his use of trichloracetic acid, as it was from one of his writings that I first learned the value of the drug in this connection.

I am glad that there is a bright prospect of discarding the cruel and unnecessary operation of cutting, or, as Dr. Perry says, the terrible hacking of the alveoli border. I have with me a few sections of bone which you will find a proof of what I said last month, and if it is your pleasure I shall be glad to pass them around for inspection.

*Dr. Jarvie.*—The etiology of this disease is certainly the most reasonable and scientific that I have listened to. It seems to accord with my own experience, as I meet with it from day to day in the mouths of patients. We have made a step in advance to-night in realizing and understanding the cause of this disease, and we will be the better able to treat it in the future. For some time I have realized that pyorrhœa alveolaris was a constitutional disease, and not a local disturbance; or rather, that the local disturbance is a manifestation of a constitutional disease. Serumic calculus is what we, as dentists, have largely to deal with as the direct cause of the loss of the teeth, and yet there is a cause beyond that, for the deposit of this serumic calculus is a result of a constitutional tendency. The paper and the discussion to-night have taken a different turn from what we heard a month ago upon this same general subject. Dr. Van Woert's paper dealt with a condition of the disease and local treatment. To-night we have gone into the cause of the disease and the constitutional treatment. Both are interesting and necessary to the proper understanding of the subject, and I cannot but feel that every gentleman in this room ought to be all the better able to treat this disease for having been present at the last two meetings of this Society. I think this is the most interesting subject we have in dentistry to-day. More teeth are lost to our adult patients from this disease than from all other causes put together.

It ought to be a most interesting subject for us to discuss, and we ought to feel that every point of information that we can get is of great value to us. I believe we shall in a large manner overcome this disease by constitutional treatment. This is not a disease that comes on in a day, or a month, or a year, as Dr. Peirce has said. It has undoubtedly been working through the system for a long time before this manifestation is made about the teeth. It will take probably as long to treat and cure constitutionally as it does to manifest itself. It would be well if the investigation of dentists and of medical men were turned in the direction of treating pyorrhœa constitutionally. We, as dentists, can probably do much to direct the attention of the medical profession to the treatment. They do not observe the results as we do. They see the same constitutional tendency manifested in gout and in rheumatism, but they do not see it in the teeth, and I believe it is manifested there much more universally among well-to-do people than it is in rheumatism or gout. In almost all cases of pyorrhœa you will find the patient of a gouty diathesis.

*Dr. Niles.*—I have been much interested in the paper of Professor Peirce and the remarks of the last speaker. I wrote a paper on the same subject about ten years ago, in the preparation of which I was able to see something of what he has proved by his experiments. I believe with him that there is a twofold deposition, first from the saliva of salts, and second from the blood or serous exudations of gum-tissue. I have never seen a deposition of lime salts on a tooth that did not have an opening from the point into the oral cavity. There are instances of musket-balls, etc., becoming encysted in the flesh. Many of our wounded soldiers in the late war have lived with balls embedded in their flesh for years; when removed they have had enormous deposits of this matter around them. I believe with the essayist and with the gentlemen who have spoken to-night, that it is a systemic condition that we especially have to deal with if we deal with the cause of the trouble. There is one point that the professor did not refer to, and that is, the possibility of the backing up of the lime salts in the system from the stoppage of the ordinary channels of excretion,—the kidneys. It has been mentioned by several writers that the salivary glands may take on themselves a vicarious action, excreting some of the excretions of the kidneys, the kidneys having become diseased, and failing in a measure to eliminate the urinary matter.

It is possible this may explain in a measure the disturbances which have been mentioned to-night.

I have seen several cases since I read the paper, which have confirmed me in my opinion. One patient was operated on, I think twice, for stone in the bladder. He had about his jaws on the inside large nodules of bone-formation encircling the inner side of the maxilla. I cleaned his teeth several times, but the deposit would soon appear again. In twenty-four hours a soft deposit would cover his teeth. The man died, and Dr. Cheever, of Boston, my neighbor, performed an autopsy on his body. He found his gall-bladder completely impacted, as well as stones in the bladder; his system throughout was charged with calcic and phosphatic matter.

Another point that was brought up in regard to the deposition on the teeth. I did not know that the urates were a large constituent of the deposit. I had not discovered it to so great an extent. I found that the phosphate of lime, the carbonate of lime, and the phosphate of magnesia made up a large part of this deposit, but I see no reason for supposing that the urates may not be present to the extent intimated in some cases.

I am very glad that I made the trip from Boston here, although when I planned it I did not know the subject was to be discussed. I believe we are on the right track. The manner of treatment with me has been doubtful so far as systemic treatment is concerned, from the fact that you can never make up a programme or describe a given course for this disease. It is so complicated with other troubles, and there are so many phases of it, that I am completely nonplussed where the question in its worst phases are to be dealt with. You can give Carlsbad or Lithia waters, etc., as has been recommended here to-night, which are supposed to keep these salts in circulation and assist in their elimination by the normal channels; but whether they do so or not to the extent desired I am not prepared to say. Physicians prescribe these remedies with supposed results named. There is one case in particular I would speak of, a young lady about twenty-four years old I treated about ten years ago, where the disease was cured and the teeth became absolutely firm. The patient says I cured the trouble, but I noticed other changes not local. There was a change in her general health for the better. I simply removed the local deposits on the teeth, and the circulation of her system changed from other causes for the better. She has kept her teeth, and they are doing good service to-day.

*Dr. Bogue.*—Dr. Michaels, of Paris, noticed that some warm water which he had in his office standing over a gas-burner collected a thicker deposit of lime at the point where the flame was than anywhere else. Acting upon this incident he made a number of experiments, and the result was this hypothesis: that the deposition of salivary calculus is largely dependent upon the elevation in temperature of the parts involved. A very slight elevation caused by inflammation suffices to cause fluids impregnated with lime to deposit this lime.

I simply repeat Dr. Michaels's statements, but they fit in precisely with what has been said this evening. Here is an observer, several thousand miles away, who by his own processes has reached much the same conclusions as are hinted at by Dr. Jarvie.

*Dr. Perry.*—My faith is growing stronger and stronger as to the cause of erosion. I want to relate the case of a lady who came to me from abroad, an old patient of mine, I think probably six months ago. I said to her instantly, "You have an excess of uric acid." She said she had not been well at all. She had neuralgia and pains in her bones and joints, and she was nervous and depressed and all out of sorts in every way. All the physicians to whom she had gone had not been able to find any cause. I said, "I am sure there is an excess of uric acid in your system. Just suggest it to your physician." I wish the gentlemen present could hear what she said to me last week in reference to what had been done for her after giving her physician that hint. She said she was a reformed woman in regard to health, and that life was worth living.

*Dr. Niles.*—I want to speak of one condition, and that is, the absence in some cases of the deposit. I have never been able to ascertain whether the deposit might not have been there at the inception of the trouble, and the chemical conditions becoming so altered to an acid condition that a deposit could not form. Has this occurred to any gentleman present?

*Dr. Perry.*—I hope we will not pass an ordinary vote of thanks to Dr. Peirce for reading this paper. If we do, I hope some gratification will be felt, if not expressed, that as the years go on he still retains his boyish enthusiasm and his interest in science. It was worth coming farther than from Boston to New York to hear this paper. It was worth going from here to Chicago. I will put my remarks in the form of a resolution.

Resolution unanimously carried.

*Dr. Peirce.*—I have had special gratification in being with you this evening. My work is not finished. It is just beginning. The opportunity that lies before us all is very important. One swallow does not make a summer, as you know, nor are the experiments of one individual sufficient to establish an hypothesis. We need to have scores of teeth examined, and these results confirmed, before we can start in on a sure basis. I have planned to occupy the winter with further investigations, for I feel that I am on the right track. It has been a pleasure to me to-night to have such a hearty response to my immature paper.

*Dr. Jarvie.*—I think a word at this time would be very appropriate. A month ago we had a paper from Dr. Van Woert on the subject of pyorrhœa alveolaris. We have had this paper from Dr. Peirce to-night, and in it he stated he had some further experiments under way. I wonder if we could not pre-empt the results of these experiments and have this matter go out before the public as coming from us. For the sake of the cause this would be wise. The effect would be better than if the papers and discussions were scattered among different bodies. I know Professor Peirce has his affiliations in Philadelphia, but possibly he might be prevailed upon to read the result of the further investigations before this Society, and we will try to make the assembly as representative of the dental profession as we can, and not confine it merely to that part that is in New York.

*Dr. Peirce.*—You must remember that a year ago I read a paper before the Odontological Society of Pennsylvania, and they may feel that it is a little infringement on their rights for me to bring this paper here to-night. I presume the subsequent investigations will probably be more needed there than here. Some Philadelphians are much averse to the theory advanced this evening, and the next paper when ready will, I think, be read in Philadelphia if at all welcome. If I am to judge from your criticisms, which have all been so kindly and considerate, it will there be most appropriate.

Adjourned.

John I. Hart, D.D.S.,
*Editor New York Odontological Society.*

